# Insulin-IGF signaling affects cell transformation in the BALB/c 3T3 cell model

**DOI:** 10.1038/srep37120

**Published:** 2016-11-16

**Authors:** Doerte Poburski, Christiane Leovsky, Josefine Barbara Boerner, Luisa Szimmtenings, Michael Ristow, Michael Glei, René Thierbach

**Affiliations:** 1 Institute of Nutrition, Friedrich Schiller University Jena, Dornburger Str. 24, 07743 Jena, Germany

## Abstract

The increased cancer mortality of diabetes type 2 patients is most likely an evidence of the tight connection between tumor development and energy metabolism. A major focus of today’s research is still the identification of key proteins of both diseases and the development of corresponding inhibitors. In this study we combined the two-stage BALB/c-3T3 cell transformation assay (BALB-CTA) with the IR/IGF-1R inhibitor OSI-906 (linsitinib) and analyzed alterations in protein activity and energy parameters in non-transformed as well as transformed cells. OSI-906 successfully inhibited the phosphorylation of IR/IGF-1R and decreased cell growth in non-transformed cells. In the BALB-CTA, a permanent treatment with OSI-906 reduced cellular transformation dose-dependently, whereas a temporary treatment gave evidence for a preventive effect in the promotion phase. Furthermore, even though several key proteins were affected, it was possible to show that the phosphorylation of GSK3, Erk 1/2 and the S6 protein are not crucial for the cell foci reducing effect of OSI-906. Taken together, the BALB-CTA confirmed results of OSI-906 from animal studies and enhanced the knowledge of its mode of action. Therefore, the BALB-CTA offers the opportunity to analyze alterations in the transformation process more precisely and will be helpful to identify effective cancer treatments.

Cancer is still a major public health problem resulting in approximately 8 million cancer-related deaths per year worldwide and an estimated annual economic cost of US$ 1.16 trillion in 2010^1^. Despite decades of research, the investigations of the underlying mechanisms are still ongoing, which is critical for the development of effective treatments or to take action in preventing the causes. It is clear that cancer is a multifactorial and multistep process and more than 100 distinct cancer types as well as subtypes of tumors in different organs exist^2^. This complexity makes it hard to elucidate the origin and treatment of this harmful disease.

To study the molecular mechanisms of cancer, it is not always reasonable to conduct a human clinical trial or perform long lasting animal experiments. Basic knowledge of alterations in cellular neoplastic processes can be obtained with *in vitro* cell transformation assays (CTAs)^3^. CTAs mimic different stages of *in vivo* carcinogenesis and represent an excellent alternative to study cancer mechanisms and therapeutic options^4^. CTAs are faster as well as less expensive than animal experiments and they are able to detect both genotoxic and non-genotoxic carcinogens^5^,^6^. The BALB/c cell transformation assay (BALB-CTA) is based on the immortalized embryonic mouse fibroblasts BALB/c-3T3^7^ using the subclone A31-1-1 (BALB/c cells) by Kakunaga and Crow^8^. BALB/c cells form a monolayer culture and get contact-inhibited after reaching confluence. Upon treatment with tumor initiators and promoters in a classical two-stage cancer model, some cells are transformed and grow as morphologically aberrant foci over the monolayer of non-transformed cells^9^,^10^. Our group further improved the BALB-CTA and combined the standard protocol with a parallel treatment of substances of interest. In addition, the BALB-CTA was combined with several endpoint applications, like analysis of protein level and signaling (western blot, immunofluorescence, subcellular fractionation) as well as parameters of energy metabolism (glucose and oxygen consumption)^3^. Hence, the BALB-CTA is more than a standard toxicological method and can provide essential information regarding key proteins and their signaling during the different stages of cell transformation and can help to identify potential cancer therapeutics.

The development of cancer cells and tumors relies not only on evading cell death and growth control, but also requires adjustments in energy metabolism providing sufficient energy products for the uncontrolled cell duplication^11^. Reprogramming of energy metabolism was first observed by Otto Warburg, who showed that neoplastic cells favor glycolysis even in the presence of oxygen^12^,^13^. To compensate the poor efficiency of ATP production via aerobic glycolysis compared to oxidative phosphorylation cancer cells for example increase glucose import by upregulating glucose transporters^14^,^15^. For cancer cells, an advantage of an increased glycolysis is the supply with intermediates of glucose degradation, which are important for several biosynthesis pathways (nucleotides, lipids, NADH)^16^. Evidence for the link between energy metabolism and cancer development can also be found in epidemiological studies, which reveal an association between type 2 diabetes mellitus (T2D) and an increased risk for several cancer types^17^,^18^. On the other hand, anti-diabetic drugs like metformin appear to decrease the risk of cancer or decreases metastases^19^,^20^, although the underlying molecular mechanisms are not fully elucidated until now. Possible links between T2D and cancer seem to be amongst others a defective insulin response, resulting in insulin resistance and hyperinsulinemia as well as increased levels of bioavailable insulin-like growth factor 1 (IGF-1)^21^.

The metabolic effects of insulin and IGF-1 are mediated by their corresponding receptors, the insulin receptor (IR) and the insulin-like growth factor 1 receptor (IGF-1R). Both, the IR^22^,^23^,^24^ and IGF-1R^25^,^26^ have been found to be overexpressed in neoplastic cells and human tumors and represent a promising point of action in cancer therapy. The anti-tumor efficiency was demonstrated for example by different strategies of IGF-1R inhibition in *in vitro* and *in vivo* tumor growth models^27^,^28^. Additionally, hybrid receptors of IR and IGF-1R have been found in different cancer cells and may also play a prominent role in carcinogenesis^23^. Binding of the corresponding ligands to IR, IGF-1R or hybrid receptors stimulate downstream pathways like PI3 kinase/protein kinase B/mammalian target of rapamycin (PI3K/Akt/mTOR) and Ras/MEK/extracellular signal-regulated kinase (Ras/MEK/Erk), which influence cellular growth, apoptosis, proliferation, cell transformation and metastasis^29^,^30^,^31^. Targeting IGF-1R in human cancers seemed to be a promising approach, but the results of the clinical trials with IGF-1R inhibitors as a single agent proved to be disappointing^32^. Therefore, new strategies aim for the crosstalk between IGF-1R and other pathways and try combinations with IGF-1R inhibitors and other targeting agents like the IR or epidermal growth factor receptor (EGFR)^33^.

OSI-906 (linsitinib) is a selective dual inhibitor of the IR and IGF-1R and therefore a promising candidate for the treatment of various cancer types. OSI-906 showed anti-proliferative effects in several colorectal cancer (CRC) and non-small-cell lung cancer tumor cell lines^34^,^35^ and inhibited tumor growth in different xenograft mouse models^34^,^36^,^37^. Anti-tumor activities have been reported in phase I clinical trials with colorectal cancer and adrenocortical carcinoma^38^,^39^. Mechanistically, OSI-906 is able to impede the activation of downstream proteins of the PI3K-Akt and Ras-Erk pathway^34^. Modulation of IR and IGF-1 R signaling and the downstream target Akt is linked to the regulation of glucose metabolism (glucose transporter activation, stimulation of hexokinase and phosphofructokinase activity)^40^,^41^ and OSI-906 treatment of mice has been shown to decrease glucose uptake dose-dependently^42^.

In this study we wanted to investigate the influence of a modulation of the insulin-IGF network on non-transformed and transformed cells as well as the cellular transformation process. Therefore, we combined the IR/IGF-1 R inhibitor OSI-906 with the *in vitro* BALB-CTA and analyzed alterations in transformed foci formation. Several proteins in the IR/IGF-1 R axis are known for their involvement in tumor development and we wanted to get some insights into protein activations during transformation and OSI-906 treatment. As part of the chief mechanisms for controlling cell survival, differentiation, proliferation and metabolism we wanted to elucidate the roles of Akt and the protein Erk in cellular transformation^43^. Further downstream the mTOR complex 1 (mTORC1) and its substrates p70 ribosomal protein S6 kinase (p70S6K), ribosomal S6 protein (S6) and eIF4E-binding protein 1 (4E-BP1) have been recognized as important players in carcinogenesis^44^,^45^. Finally, to analyze the potential connection between energy metabolism and cancer mechanisms with OSI-906, we studied the role of the glycogen synthase kinase 3 (GSK3) and further parameters.

## Results

### OSI-906 affects non-transformed BALB/c cells dose-dependently

**Figure 1:**
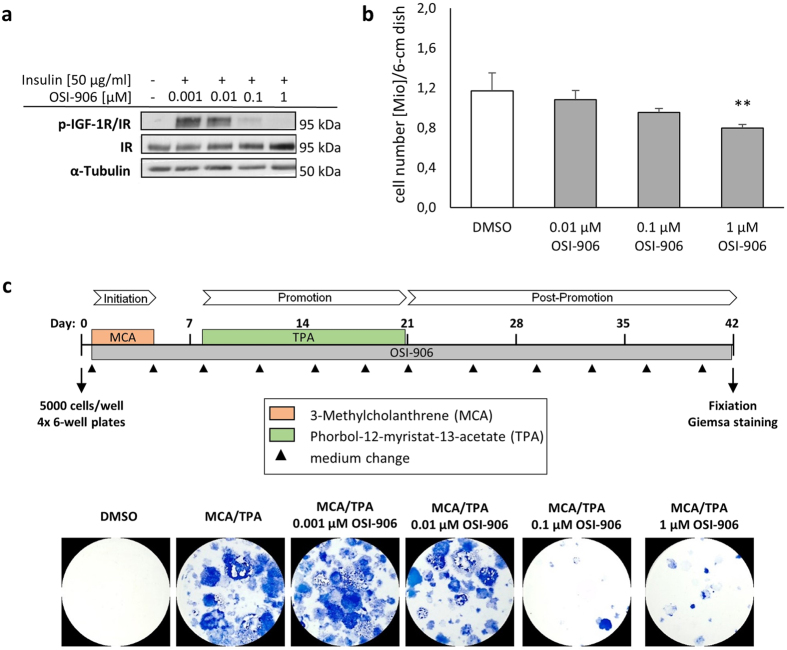
OSI-906 influences IGF-1R/IR activation, cell proliferation and transformation. (**a**) BALB/c cells were incubated for 24 h with different concentration of OSI-906 and 5 min with 50 μg/ml insulin. After protein extraction immunoblot analysis showed a dose-dependent decrease of the IGF-1R and IR phosphorylation. Shown are representative blots of 2 independent experiments. (**b**) BALB/c cells were treated with 0.01, 0.1 and 1 μM OSI-906 for 48 h. Afterwards cell viability was determined using the trypan blue exclusion test. OSI-906 causes a significant growth inhibition at 1 μM. Results indicated are mean + SD of the viable-cells of 3 independent experiments (no non-viable cells were detected). Statistical differences are displayed as **(p < 0.01) according to a one-way ANOVA (post-hoc Dunnett). (**c**) BALB-CTAs were performed with 0.5 g/μl 3-Methylcholanthrene (MCA) on day 1–4 (initiation phase) and 0.3 μg/μl 12-O-Tetradecanoyl-phorbol-13-acetate (TPA) on day 8–21 (promotion phase). Different concentrations of OSI-906 were applied during the whole assay from day 1–42. After 42 days cells were fixed with methanol and transformed cell foci were visualized by Giemsa staining (blue colored). Presented are selected pictures of 2 independent experiments, each with 4 replicates. OSI-906 shows a dose-dependent decrease in cell transformation.

OSI-906 is a selective inhibitor of the IR and the IGF-1R and we first wanted to monitor acute effects of a treatment in non-transformed BALB/c-3T3-A31-1-1 cells used for the transformation assay. After 24 hours treatment, OSI-906 decreased the phosphorylation of the IR/IGF-1R in a dose-dependent manner with no effect for 0.001 μM and a complete inhibition for 1 μM (Fig. 1a). Furthermore, OSI-906 treatment revealed a growth inhibition in BALB/c cells after 48 hours treatment with 0.1 and 1 μM (Fig. 1b) and showed no difference in oxygen consumption for 1 μM (Supplementary Fig. 1). On the basis of the described experiments, 1 μM OSI-906 was defined as the working concentration for the CTAs, with the most impact on IR/IGF-1R signaling and a tolerable growth inhibition.

### OSI-906 suppresses cellular transformation

To test whether a decreased insulin/IGF signaling influences cellular transformation a two-stage BALB-CTA was performed. For this, cells were seeded into 6-well plates and maintained over 6 weeks, with medium changes every third/fourth day. After treatment with the tumor initiator MCA (treated on day 1 to 4) followed by the tumor promoter TPA (day 8–21), transformed cells started to grow over the normal monolayer and piled up to cell foci. The morphological aberrant foci appear blue colored after Giemsa staining and can be distinguished from the monolayer according to recommended criteria^46^. Figure 1c displays the result of a BALB-CTA cultured with MCA/TPA and in addition with different concentrations of OSI-906 given from day 1 to 42. DMSO was used as a solvent control and induced no blue colored cell foci, whereas MCA/TPA treatment as the positive control led to a massive development of transformed foci. An additional treatment with OSI-906 decreased cellular transformation, most effectively with 0.1 and 1 μM. For more detailed results of the foci scoring see Supplementary Fig. 2. The anti-transformation potential of OSI-906 is not restricted to MCA/TPA, but could also be detected with other tumor initiators and promoters (Supplementary Fig. 3). Additional experiments with colchicine were performed to evaluate the effect of a reduced cell growth on the development of transformed foci. Although concentrations of colchicine more effectively decreased cell proliferation compared to OSI-906, the amount of developed foci was still higher (Supplementary Fig. 4).

**Figure 2:**
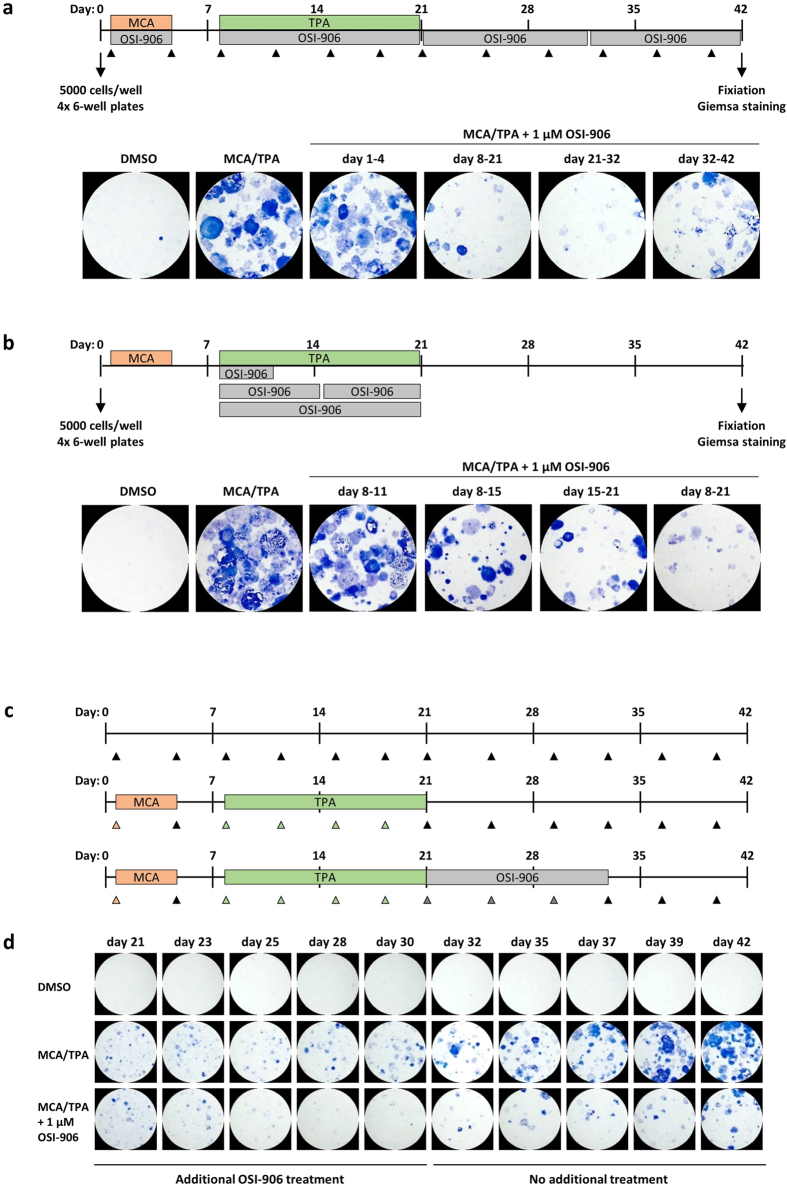
OSI-906 shows preventive and therapeutic activities. (**a**) BALB-CTAs were performed with additional treatment of 1 μM OSI-906 on day 1–4 (initiation phase), on day 8–21 (promotion phase) and on day 21–32 and 32–42 (post-promotion phase). Presented are selected pictures of 3 independent experiments, each with 4 replicates. OSI-906 was able to decrease cellular transformation already during the promotion phase and shows a therapeutic effect in the later stages of transformation. (**b**) A BALB-CTA with 4 replicates was performed to analyze the promotion phase of transformation in more detail. 1 μM OSI-906 was applied on day 8–11, 8–15, 15–21 and during the whole promotion phase on day 8–21. OSI-906 reduced the formation of cell foci (blue colored) after more than 3 days of treatment in the promotion phase. (**c**,**d**) A BALB-CTA with DMSO (negative control), MCA/TPA (positive control) and MCA/TPA with 1 μM OSI-906 on day 21–32 was performed, to clarify the OSI-906 effect on the cell foci formation in a time response experiment. Therefore, between days 21–42 one 6-well plate was fixed and Giemsa stained every second/third day. OSI-906 decreases the already developed cell foci from day 21 on and prevents the enormous increase in cellular transformation compared to the MCA/TPA positive control.

OSI-906 is described as a promising substance for cancer treatment^34^ and we wanted to transfer this application to the BALB-CTA. Therefore, we applied 1 μM OSI-906 in various stages of transformation, initiation day 1–4, promotion day 8–21 and post-promotion day 21–32 and 32–42, to see whether OSI-906 is able to prevent cells from malignant transformation or has a therapeutic effect on the already transformed cell foci (Fig. 2a). It turned out that foci formation was reduced when OSI-906 was admitted on day 8–21 (promotion phase), day 21–32 and day 32–42 (post-promotion-phase). No effect was detected when OSI-906 was applied only in the initiation phase of cellular transformation (day 1–4), which was not changed by an even longer treatment from day 1–8 (data not shown). A detailed analysis of OSI-906 usage in the promotion-phase of the BALB-CTA revealed that a short-term treatment (day 8–11) has no visible effect, but a treatment longer than six days (day 8–15; day 15–21; day 8–21) decreases the development of blue colored foci (Fig. 2b). To visualize the therapeutic properties of OSI-906 on already transformed BALB/c cells, a single more complex experiment was conducted. In a time response analysis (Fig. 2c) of the post-promotion phase (day 21–42) cells were fixed and stained every second/third day after treatment with MCA/TPA followed by OSI-906 (day 21–32). The amount of blue colored foci after initiation and promotion phase (day 21) started at a similar point in the MCA/TPA and MCA/TPA + OSI-906 group (Fig. 2d). Afterwards, transformed cells treated with MCA/TPA alone, began heavily to proliferate and piled up to cell foci over time. In contrast, while cells were additionally treated with 1 μM OSI-906 (day 21–32) the transformed cells did not continue proliferation and foci development, but rather showed a reduction. After completion of the OSI-906 treatment (day 32–42) the foci development increased again and OSI-906 showed no prolonged effectiveness.

### Several key proteins of the insulin/IGF axis are altered by OSI-906 treatment

**Figure 3:**
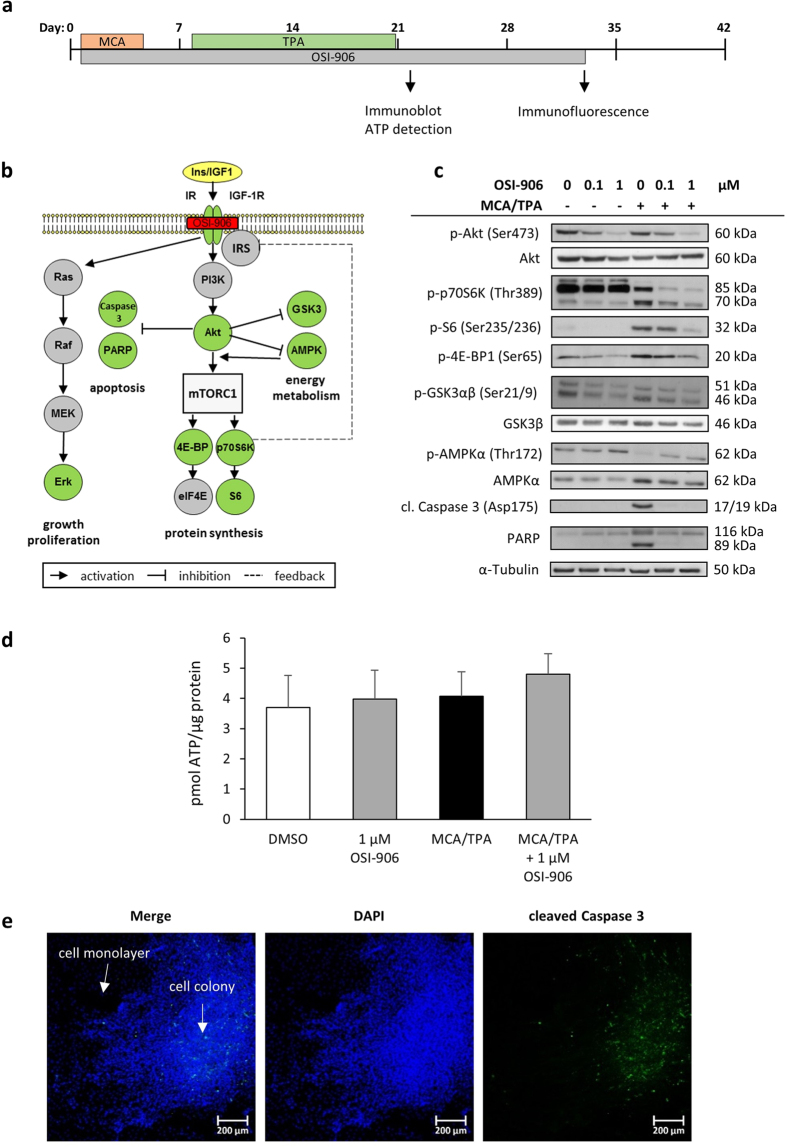
Inhibition of the IR-IGF-1R axis by OSI-906 affects several downstream pathways. (**a**) OSI-906 was applied to the BALB-CTA on day 1 to 33. Endpoint applications like ATP measurements and immunoblots were conducted on day 22, while immunofluorescence analysis were performed on day 33. (**b**) OSI-906 is a selective dual inhibitor of the Insulin and IGF-1 receptor and influences cell signaling via the PI3K/Akt and MEK/Erk pathways. Thus, OSI-906 can affect cell proliferation and growth, protein synthesis, apoptosis and cellular energy metabolism. (**c**) BALB-CTAs with different concentrations of OSI-906 + /− MCA/TPA were performed and cells were harvested on day 22 (end of promotion phase). Protein samples were analyzed with immunoblot for several target proteins. Illustrated are selected pictures of 3 independent experiments. (**d**) BALB-CTAs were performed with 1 μM OSI-906 + /− MCA/TPA. On day 22 of the transformation assay ATP content of the cells was measured and normalized to protein. Results indicated are mean + SD of 3 independent experiments. Statistical differences were calculated with a one-way ANOVA. ATP content of the cells appeared to be not significantly different between the 4 groups. (**e**) BALB-CTAs with MCA/TPA were performed until day 33. Cells were fixed and stained with DAPI as well as cleaved Caspase 3. Multilayer growth of the transformed cells can be visualized by DAPI staining and differentiation between cell monolayer and foci is possible. Presented are selected pictures of 3 independent experiments. The cleaved Caspase 3 signal is only visible inside the cell foci and not in the surrounding monolayer. Since OSI-906 treatment led to hardly transformed foci, the apoptosis signal cannot be detected with immunofluorescence.

Besides to the impact of OSI-906 on cellular transformation, we were able to show changes in the protein activity of targets within the insulin-IGF axis, helping to better understand cell transformation processes. A BALB-CTA was performed with and without MCA/TPA as well as 0.1 and 1 μM OSI-906 (day 1–22) until day 22, where transformed cells start to grow over the normal monolayer (Fig. 3a). Since OSI-906 inhibits the phosphorylation of the IR/IGF-1R, the activation of downstream proteins of the PI3K-Akt and Ras-Erk pathway can be impeded (Fig. 3b). Immunoblot analysis on day 22 showed, that phosphorylation of Akt, S6, 4E-BP1 and GSK3 seems to decrease dose-dependently with OSI-906, when added additionally to the MCA/TPA treatment (Fig. 3c). This also applies for the phosphorylation of both isoforms of the S6-kinase 1, p70S6K (bottom band) and p85S6K (middle band), while unspecific signals (upper band) are detected by the same antibody too. In contrast, the phosphorylation of the energy sensor AMPK (AMP-activated protein kinase) increased when OSI-906 was applied to the culture medium in combination with MCA/TPA. An increase in p-AMPK is usually a signal of energy-consuming processes and a high demand of ATP^47^, which we wanted to confirm by measuring the ATP content of the cells (Fig. 3d). After 22 days of the BALB-CTA we found no significant difference between cells treated with either OSI-906 and/or MCA/TPA. The same result was found in measurements of oxygen consumption (Supplementary Fig. 1), where no significant differences were observed. For the apoptotic marker cleaved Caspase 3 and its main target PARP (shown as full length and cleaved PARP) we detected a huge immunoblot signal only in the MCA/TPA treated cells (Fig. 3c). This observation was also checked by immunofluorescence, to distinguish between transformed and non-transformed cells. After 33 days of a BALB-CTA it was possible to differentiate by DAPI staining between the cell monolayer and the transformed cell foci (Fig. 3e). Thus, the signal of Caspase 3 cleavage (green colored) could be assigned to the cell foci itself and was not located in the normal cell monolayer. Because there were almost no foci after DMSO or OSI-906 treatment, signals of Caspase 3 cleavage could not be detected (data not shown). The level of Erk 1/2 phosphorylation (data not shown) was not consistently modified in several independent experiments and therefore considered to be not causal for the OSI-906 outcome.

### GSK3 and S6 are not relevant for the foci formation in the BALB-CTA

**Figure 4:**
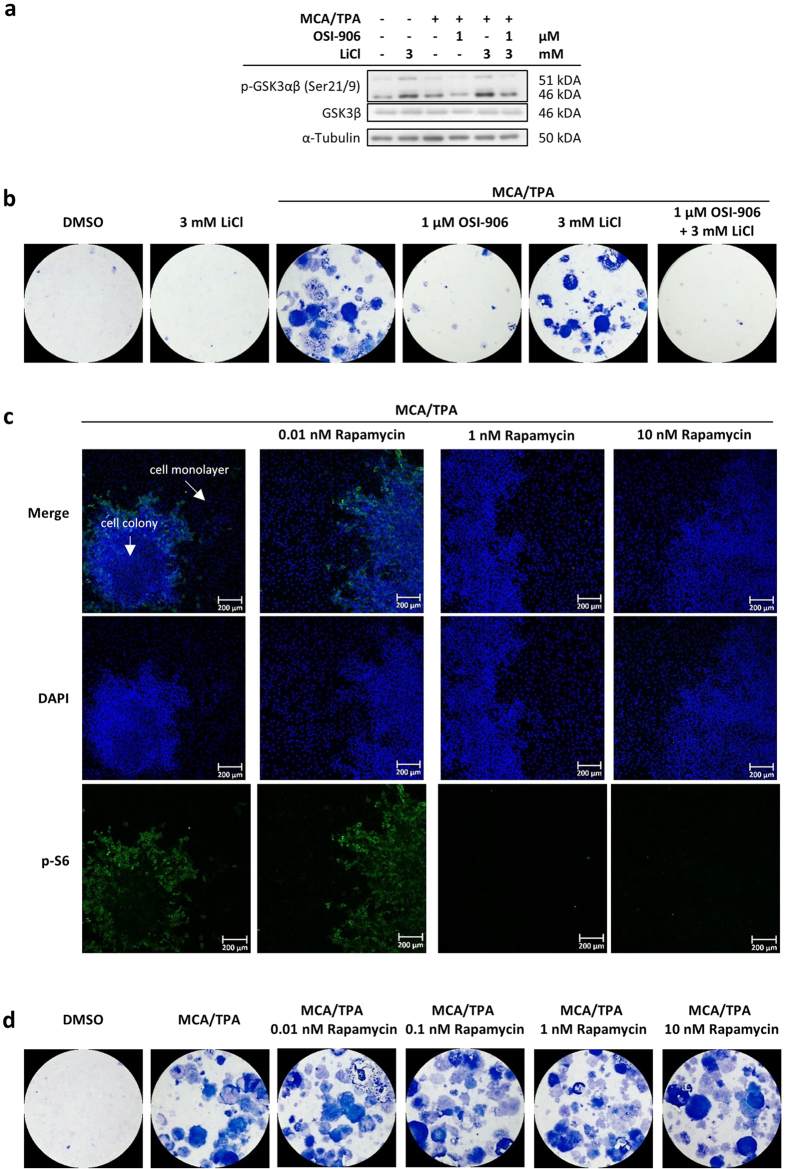
GSK3 and S6 are not causative for the OSI-906 dependent decrease in cell transformation. (**a**,**b**) A selected concentration of the IR/IGF-1R inhibitor OSI-906 and the GSK3 inhibitor lithium chloride (LiCl) were used separately and in combination in the BALB-CTA. Proteins were obtained after 22 days of treatment (n = 3) according to the standard cell transformation protocol. Furthermore, cells were fixed and Giemsa stained after 42 days of the transformation assay (n = 3). OSI-906 decreased the inhibitory GSK3 phosphorylation (GSK3 is active) compared to MCA/TPA and showed almost no cell foci (blue colored). LiCl increased the inhibitory GSK3 phosphorylation (GSK3 is inactive) and shows several transformed cell foci. The combination of both inhibitors led to a similar p-GSK3 signal to the MCA/TPA control, but no cell foci could be found compared to the great amount in the positive control. (**c**) BALB-CTAs with MCA/TPA and different concentrations of the mTORC1 inhibitor rapamycin were performed until day 33. Cells were fixed and stained with DAPI (blue) and p-S6 antibody (green). Presented are selected pictures of 3 independent experiments. Several cell foci could be found in all treatment groups (see DAPI signal). The p-S6 signal was detected at the edge of the MCA/TPA treated cell foci, which disappears with higher rapamycin concentrations (1 and 10 nM). (**d**) BALB-CTAs with different concentrations of Rapamycin and MCA/TPA were performed until day 42. Presented are selected pictures of 3 independent experiments, each with 4 replicates. Rapamycin showed no change in cellular transformation compared to the MCA/TPA positive control.

Based on the immunoblot data, the phosphorylation of GSK3 was also found to be altered with OSI-906 treatment (Fig. 3c). GSK3 is tied to proliferation as part of the canonical Wnt/beta-catenin pathway and is able to participate in different apoptotic pathways^48^. To investigate whether GSK3 plays an important role in the OSI-906 dependent decrease in cell transformation, we conducted BALB-CTAs with the GSK3 inhibitor lithium chloride (LiCl) and analyzed protein samples on day 22 additional to the staining of transformed foci with Giemsa on day 42. OSI-906 decreased the inhibitory GSK3 phosphorylation (GSK3 is active) compared to MCA/TPA alone (Fig. 4a) and led to hardly no cell transformation (Fig. 4b). A selected concentration of LiCl (3 mM) per se increased the inhibitory phosphorylation of GSK3 (GSK3 is inactive) and still led to a lot of blue colored foci. The combination of OSI-906 and LiCl exhibits a similar level of GSK3 phosphorylation as the MCA/TPA control, but nevertheless suppresses cell transformation successfully. Hence, GSK3 phosphorylation seems to be not relevant for the cell transformation process.

The mTORC1 is able to influence protein synthesis via its downstream targets p70S6K, the ribosomal S6 protein and 4E-BP1^45^. Considering that OSI-906 treatment reduced the phosphorylation of these proteins (Fig. 3c), we suspected that mTORC1 may be relevant for the foci formation. Therefore, we combined a BALB-CTA with the mTORC1 inhibitor Rapamycin and monitored the S6 phosphorylation by immunofluorescence on day 33 (Fig. 4c). When MCA/TPA or 0.01 nM Rapamycin in addition were applied to the culture medium from day 1 to 33, the S6 phosphorylation (green colored) could be recognized in the cell foci itself and not in the normal cell monolayer. Interestingly, when higher concentrations of Rapamycin (1 and 10 nM) were used, the phosphorylation of S6 was not detectable anymore, although numerous transformed foci existed (Fig. 4d). Consequently, mTORC1 signaling via S6 is not involved in cellular transformation in the BALB-CTA and cannot be responsible for the OSI-906 dependent decrease in foci formation.

## Discussion

The insulin/IGF network is tightly connected to cancer development, because it mediates cell growth, proliferation, differentiation and participates in metabolic activities^29^. The corresponding receptors of this network, the IR and IGF-1 R, were found to be overexpressed in various cancer types and an inhibition of these receptors proved to be effective against tumor growth^23^,^49^. OSI-906 is a potent small-molecule dual IR/IGF-1 R kinase inhibitor, which was first described by M. J. Mulvihill and colleagues^34^. OSI-906 showed anti-proliferative activities *in vitro* as well as anti-tumor efficiency *in vivo* so far, but failed to increase the overall survival of patients with adrenocortical carcinomas in a phase 3 trial^50^. To understand the mode of action of OSI-906 more precisely, we wanted to analyze some mechanisms contributing to its therapeutic properties. Furthermore, it was important to get some insights into alterations of the insulin/IGF network during cellular transformation, to try to develop new approaches against cancer in the future.

The BALB-CTA, originally developed as a standard toxicological method for chemical risk assessment, proves to be a suitable *in vitro* method, which mimics the initiation as well as the promotion phase of carcinogenicity. In our previous publication we already demonstrated how the BALB-CTA can be successfully combined with several endpoint applications to be used for mechanistic cancer research^3^. To study the dysregulation of an insulin/IGF-1 signaling we combined OSI-906 with the two-stage BALB-CTA. First of all, we wanted to confirm already described effects of OSI-906 in cancer cell lines with the non-transformed BALB/c cell line. As shown before in cancer cells^34^,^37^, OSI-906 was able to inhibit the phosphorylation of the IR and IGF-1 R dose-dependently and reduced cell proliferation of non-transformed BALB/c cells. Thereby, we confirmed 1 μM OSI-906 as an effective concentration. Initial cell transformation experiments with the BALB-CTA showed that OSI-906 decreased the formation of transformed foci dose-dependently, thereby supporting results from other groups in cancer cells^34^,^35^ and nude xenograft models^36^,^37^. Furthermore, when OSI-906 was applied only in specific phases of the transformation process, a preventive effect in the promotion phase as well as a therapeutic effect in later stages of carcinogenesis were discovered for the first time. Additional experiments with colchicine proved that the reduction in cell transformation by OSI-906 is not caused by its decline in cell proliferation.

To see whether an inhibition of the IR and IGF-1 R signaling by OSI-906 treatment has an impact on downstream pathways during cellular transformation, we analyzed protein samples on day 22 of the BALB-CTA (after initiation and promotion phase). Akt represents a central protein of the IR/IGF-1 R pathway, which targets several molecules of the tumor development machinery, like cell proliferation, cell metabolism and cell death through apoptosis^51^. Activated Akt can promote cell survival by suppressing proapoptotic functions of downstream targets^52^ and a decrease in Akt activity by OSI-906 may be important for the decline in cellular transformation. In several cancer cell lines it was demonstrated before that the IR/IGF-1 R inhibitor OSI-906 is able to prevent the activation of downstream targets like Akt^34^,^42^,^53^. This was confirmed in our BALB-CTA, where Akt phosphorylation was decreased dose-dependently by OSI-906 in both the non-transformed as well as the transformed BALB/c cells. It is known that an overexpression of the three different Akt isoforms can promote proliferation of cancer cells and they have been found deregulated in several cancers^54^. Whether the overall Akt activity rather than the phosphorylation of specific Akt isoforms is crucial for the transformation process has to be elucidated further.

A parallel pathway downstream of IR/IGF-1R is the Ras-Erk signaling cascade. Erk 1/2 is a mitogen activated protein kinase (MAPK) activated by the Ras GTPase and the following protein kinases Raf and MEK. Upon activation of this pathway by mitogens, growth factors or cytokines, ERK 1/2 acts as a transcription factor controlling several genes involved in cell survival, cell cycle progression and cell proliferation^55^. Therefore it is not surprising that the Erk pathway is often deregulated in numerous cancers and the development of inhibitors targeting members of this pathway seem to be an important therapy strategy^56^. MCA/TPA as well as a treatment with OSI-906 showed no consistent alteration of the Erk 1/2 phosphorylation in the BALB-CTA, which goes along with opposed effects reported in the literature^34^,^35^,^53^. Due to the tight crosstalk of the PI3K-Akt and Ras-Erk pathways^43^ and their positive as well as negative regulations on one another it is hard to distinguish between additive effects and this may be an explanation for the irregular results. Since Erk phosphorylation was not consistent between several independent experiments, it was considered to be not causal for the OSI-906 foci reduction.

The mTORC1 is located downstream of the Ras-Erk and PI3K-Akt signaling pathway and its deregulation has been found in various human cancer types^44^,^57^. Since mTORC1 is recognized as an important player in the pathogenesis of cancer a lot of time and effort have been invested to understand and suppress mTORC1 signaling as an anti-tumor strategy^58^. The mTORC1 controls several processes required for cancer development, like protein synthesis, proliferation, cell survival and energy metabolism by its downstream targets 4E-BP1, p70S6K and its substrate S6^45^. A phosphorylation of 4E-BP1 leads to the release of eIF4E and the initiation of translation, which seems to be decreased with OSI-906 treatment in non-transformed as well as transformed BALB/c cells. Inhibition of the IR/IGF-1 R signaling by OSI-906 also diminished the activation of p70S6K and its substrate S6 and therefore impeded protein synthesis. Elevated mTORC1 signaling is a common event in human tumors and was previously shown in breast, colorectal, hepatocellular or lung cancer^44^. The low mTORC1 signaling activity found with OSI-906 treatment in the BALB-CTA might therefore contribute to the reduction in transformed foci. With the help of established immunofluorescence analysis during the BALB-CTA it was possible to show, that an inhibition of the S6 protein by the well-known mTORC1 inhibitor Rapamycin^59^ was not able to drive foci formation down. Therefore, we concluded that the S6 protein is not relevant for the OSI-906 effect on cell transformation which was also demonstrated by other groups^60^,^61^. More evidence in the literature ascribes 4E-BP1 as a key factor in tumor formation^45^,^62^ and we found a reduced phosphorylation after OSI-906 treatment. So far, described effects of 4E-BP1 remain controversial, but 4E-BP1 may be a promising target for further investigations.

GSK3 activity is crucial for the beta-catenin degradation or stabilization in the Wnt/beta-catenin signaling pathway, which stimulates transcription of cancer-associated genes^63^. Thus, it made sense to look closer on the impact of GSK3 on cell foci formation and alterations occurring when IR/IGF1R signaling was inhibited. Cells transformed with MCA/TPA showed an inactivation of GSK3 activity (increase of phosphorylation), which was the opposite when OSI-906 was applied either in the non-transformed or transformed cells. The unique setup and short period of time of the BALB-CTA made it possible to adjust GSK3 phosphorylation with the IR/IGF-1R inhibitor OSI-906 and the GSK3 inhibitor LiCl. These experiments indicated, that the amount of cell transformation is independent of GSK3 phosphorylation and on this account, not relevant for the OSI-906 effect. However, besides to the involvement of GSK3 in tumor development, it is also a key enzyme in glycogen synthesis and the overall IGF-1 R/IR signaling is crucial for regulating glucose metabolism^23^. Due to the findings that OSI-906 decreased acute glucose consumption dose-dependently in starved NCI-H292 cells^42^ and that C57BL/6 J mice showed a deteriorated glucose tolerance^64^ we examined markers of energy metabolism in the BALB-CTA, too. Although we were able to detect a slightly elevated phosphorylation of the energy sensor AMPK after OSI-906 treatment, we found no difference in ATP content or oxygen consumption on day 22 of the BALB-CTA. Accordingly, we could not prove that a deregulation of energy metabolism plays an important role in the OSI-906 outcome.

Alongside to the involvement of the energy metabolism as a central hallmark of carcinogenesis we furthermore looked for markers of apoptosis^11^. There are opposed reports on apoptosis after treatment with OSI-906 in the literature, ranging from increased apoptosis in GEO CRC cells and OSI-906 treated xenograft tumors^37^ to no change in several CRC cell lines^35^. Our studies on day 22 of the BALB-CTA revealed that cleavage of Caspase 3 and also cleavage of its main target PARP was upregulated in the transformed cells, which was withdrawn by OSI-906 treatment. This observation should be investigated further by immunofluorescence for a better discrimination. Thereby, cleavage of Caspase 3 could be attributed only to the transformed cell foci and appeared not in the surrounding cell monolayer of non-transformed cells. As a matter of fact there were almost no transformed foci after OSI-906 treatment and therefore no detectable apoptosis signal. Whether apoptosis plays an important role in the OSI-906 dependent inhibition of transformation cannot be finally clarified. Further flow cytometry experiments may identify early and late apoptotic events in both non-transformed and transformed cells after OSI-906 treatment as well as the influence on cell foci formation.

The BALB-CTA proved to be an excellent tool to investigate new cancer therapeutics and explore involved cancer mechanisms in an *in vitro* two-stage carcinogenesis cell model. Using the IR/IGF-1R inhibitor OSI-906, we analyzed alterations in non-transformed and transformed cells and the overall impact on the transformation process. Our results confirmed that OSI-906 reduces cellular transformation. Additionally, we were able to present evidence for a preventive effect in the promotion phase. Although it was not possible to elucidate the complete mechanism of the OSI-906 effect on cellular transformation, it was possible to show that Erk 1/2, GSK3 and the ribosomal S6 protein are not crucial for the reduction in foci formation. On the other hand, the central node Akt of the IR/IGF-1R downstream pathways seems to be mostly effected and remains as a promising target for further cancer therapeutics.

In conclusion, the BALB-CTA offers the opportunity to analyze alterations of key proteins and energy parameters in non-transformed compared to transformed cells and their contribution to cancer initiation or promotion. In future, the BALB-CTA will be helpful to explore tumor therapeutic drugs and understand their mode of action more precisely, to identify effective cancer treatments.

## Methods

### Cell culture

BALB/c-3T3-A31-1-1 cells from Hatano Research Institute of Japan were kindly provided by Dr. A. Poth (Harlan Cytotest Cell Research GmbH, Roßdorf). Cells were cultured in DMEM/Ham’s F-12 (Biochrom #T481-10) containing 3 g/l D-glucose, 5% fetal bovine serum and 1% penicillin/streptomycin in a humidified incubator (37 °C, 5% CO_2_, 95% humidity). Only sub-confluent cells (about 70% confluence) between the passages 20 to 40 were used for the BALB-CTA. Cells were routinely tested for mycoplasma (the last time in March 2016).

### BALB-CTA

#### Two-stage protocol

A two-stage BALB-CTA was performed as previously described^3^. In detail, 5000 cells per well were seeded as 4 replicates into Corning® Primaria™ 6-well plates (VWR #734-0077) and cultured under standard conditions (37 °C, 5% CO_2_, 95% humidity) for 42 days, to obtain sufficient foci formation. In order to create the same conditions for analysis in the different transformation stages we used only DMEM/HAM’s F-12 medium during the whole experiment. Medium changes took place every third or fourth day (see pattern of treatment, Fig. 1c), with additional treatment of 0.5 μg/ml 3-Methylcholanthrene (MCA, Sigma #213942, dissolved in DMSO) as tumor initiator on day 1 and 0.3 μg/ml 12-O-Tetradecanoyl-phorbol-13-acetate (TPA, Sigma #79346, dissolved in DMSO) as tumor promotor on the days 8, 11, 15 and 18. The IR/IGF-1 R inhibitor OSI-906 (Active Biochem #A-1058, dissolved in DMSO) was applied either completely (every medium change from day 1 to 42) or in different phases of the transformation assay (initiation: day 1-4; promotion: day 8–11; day 8–15; day 15–21; day 8–21; post-promotion: day 21–32; day 32–42) in concentrations between 0.001 to 1 μM. 1 μg/ml Methylnitronitrosoguanidine (MNNG, TCI #M0527, dissolved in DMSO) was applied to the BALB-CTA as an alternative tumor initiator according to Ao *et al*.^65^ and 20 μg/ml insulin (Sigma #I0516) as an alternative tumor promotor on the basis of Maeshima *et al*.^66^. The GSK3 inhibitor lithium chloride (LiCl, Sigma #62476, dissolved in water) was first tested in concentrations over 10 mM according to Vidal *et al*.^67^, but clearly altered cellular growth of the BALB/c cells (data not shown). Due to the fact that 3 mM still had an effect on GSK3 activity, this concentration was chosen for the CTA. The mTORC1 inhibitor Rapamycin (US Biological #R1137-01, dissolved in DMSO) was tested for its growth inhibition and transformational potential on BALB/c cells (data not shown) and concentrations from 0.01 to 10 nM were selected for the experiments. For all experiments DMSO served as a solvent control, has been adapted for all treatments and was below 0.06% (except for BALB-CTA’s with Rapamycin: <0.15%). Unless stated otherwise, BALB-CTA’s were performed with 4 technical replicates in 3 independent experiments.

Additional analysis like immunoblot or energy parameter measurements can be realized throughout the BALB-CTA. Most experiments were performed on day 22, which was found to be the best time point to analyze alterations after the promotion phase of transformation. Only immunofluorescence experiments were conducted on day 33 to obtain a sufficient foci size for reliable results.

#### Fixation and foci scoring

After 42 days cells were washed twice with PBS, fixed with PBS/methanol (1:1) for 3 min and 100% ice-cold methanol for 10 min and washed twice with methanol. For analyzing of the tumor forming potential, cells were stained with Giemsa (AppliChem #A0885) and transformed cell foci appear blue colored. Giemsa staining was conducted as followed: 3 min Giemsa solution (1 ml/well), adding deionized water (3 ml/well) for further 3 min, 5 times washing with tab water followed by 5 × 10 min washing with deionized water on the plate shaker.

Transformed foci were manually scored using a stereomicroscope (Motic SMZ140) by two experienced persons. Type 3 foci were scored on the basis of the recommended photo catalogue^46^ and the following criteria: (i) deep basophilic staining, (ii) spindle-shaped cells, (iii) multilayer growth, (iv) random cell orientation and (v) invasive growth of cells at the edge of foci. Foci with a diameter less than 2 mm were not included into the scoring.

### Protein extraction and immunodetection

Protein samples were prepared by lysing (Cell signaling lysis buffer, #9803) and sonicating (Bandelin Sonopuls, Berlin, Germany) of the cells and quantified according to Bradford’s method^68^. SDS-PAGE was performed with a 10% (>40 kDa) or 16% (<40 kDa) gel and 30 μg protein extract per lane. The separated proteins were transferred to a polyvinylidene fluoride membrane by semi-dry western blotting, followed by incubation with different antibodies. The following antibodies by Cell Signaling were used with a dilution of 1:1000: p-Akt (#9271), Akt (#9272), p-p70S6K (#9205), p-S6 (#2211), p-4E-BP1 (#9451), p-GSK3αβ (#9331), GSK3β (#9315), cleaved Caspase 3 (#9664), PARP (#9542), p-AMPK (#2531), AMPK (#2532), anti-rabbit (#7074) as well as α-Tubulin by Sigma-Aldrich (#T9026). All protein samples were generated for immunoblot detection in 3 independent experiments.

### Trypan blue viability test

Cells were seeded into 6-cm culture dishes (75.000 per dish), attached overnight and were than treated for 48 h with different concentrations of OSI-906. Afterwards cells were trypsinized, diluted with trypan blue and viable as well as non-viable cells were counted in a Neubauer chamber. The trypan blue viability test was performed with 3 technical replicates in 3 independent experiments.

### Detection of ATP

Cells were treated according to the standard cell transformation protocol in 24-well plates. After 22 days ATP content of the cells was quantified by the CellTiter-Glo Luminescent Cell Viability Kit (Promega Cat.: G7571). Protein content was determined by the bicinchoninic acid assay (Thermo Scientific #23222/23224)^69^ for normalization. For ATP detection 3 independent experiments with 6 technical replicates were conducted.

### Confocal immunofluorescence microscopy

Cells were treated according to the standard cell transformation protocol in a μ-slide 8-well chamber (ibidi #80826). After 33 days, a sufficient cell foci size was achieved and the cells were washed with PBS and fixed for 20 min with 2% para-formaldehyde and 10 min with 0.1% Triton® X-100. After washing with 1% bovine serum albumin as blocking solution (BSA) cell nuclei were stained with DAPI (Sigma D9542). Afterwards the primary antibody cleaved Caspase 3 (Cell Signaling #9664) or p-S6 (Cell Signaling #2211) was applied, followed by the secondary antibody anti-rabbit (Cell Signaling #4412). Washing 2x with BSA was done between each incubation. Finally cells were covered with ibidi mounting medium (ibidi #50001) and analyzed using a Zeiss LSM 780 confocal microscope. Immunofluorescence analysis were performed 3 times.

### Statistical analysis

Results of the trypan blue viability test (3 independent experiments with 3 technical replicates each) and ATP measurements (3 independent experiments with 6 technical replicates each) were tested for normal distribution and homogeneity of variances with the IBM SPSS software. Statistical differences were calculated according to a one-way ANOVA with an additional Dunnett post-hoc test for the trypan blue viability test.

Experimental data of the BALB-CTA foci scoring were analyzed by a one-way ANOVA with additional Bonferroni post-hoc test. Mean number of foci/well were tested for significant differences to the solvent control (DMSO) as well as to the positive control (MCA/TPA).

## Additional Information

**How to cite this article**: Poburski, D. *et al*. Insulin-IGF signaling affects cell transformation in the BALB/c 3T3 cell model. *Sci. Rep.* **6**, 37120; doi: 10.1038/srep37120 (2016).

**Publisher’s note**: Springer Nature remains neutral with regard to jurisdictional claims in published maps and institutional affiliations.

## Supplementary Material

Supplementary Information
