# Supplementary material for: Insulin-IGF signaling affects cell transformation in the BALB/c 3T3 cell model
Source: Sci Rep. 2016 Nov 16;6:37120. doi: 10.1038/srep37120 (PMC5111065; doi:10.1038/srep37120)
Supplement: Supplementary Information [file srep37120-s1.doc]

**Insulin-IGF signaling affects cell transformation in the BALB/c 3T3 cell model**

Doerte Poburski^1^, Christiane Leovsky^1^, Josefine Barbara Boerner^1^, Luisa Szimmtenings^1^, Michael Ristow^1,2^, Michael Glei^1^, René Thierbach^1*^

^1^ Institute of Nutrition, Friedrich Schiller University Jena, Dornburger Straße 24, 07743 Jena, Germany

^2^ Current address: Energy Metabolism Laboratory, ETH Zurich, Schorenstrasse 16, 8603 Schwerzenbach, Switzerland

**Supplement Figure 1**

**
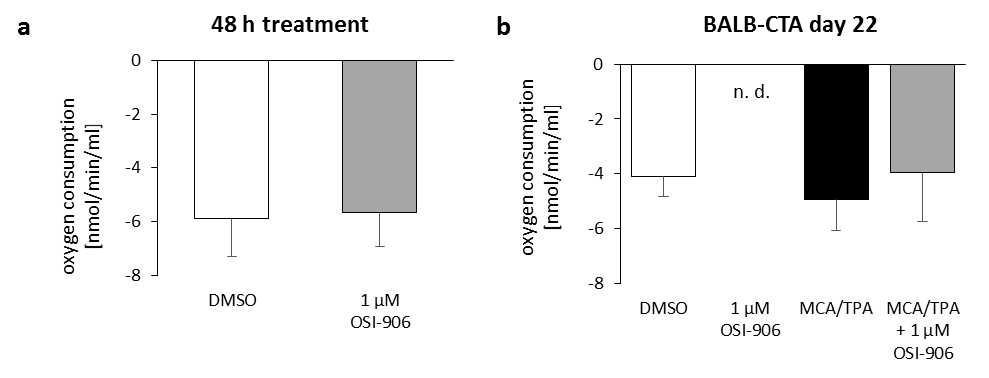
**

**Fig. S1: OSI-906 treatment shows no change in oxygen consumption**

**a:** BALB/c cells were treated with 1 µM of the IR/IGF-1R inhibitor OSI-906 for 48 hours. Afterwards 2 Mio cells/ml were applied into a clark-type electrode chamber and oxygen consumption was measured. Results indicated are mean + SD of 4 independent experiments with 4 technical replicates each. Statistical differences were calculated with a Student's t-test (for paired samples) and appeared to be not significant.

**b:** BALB-CTAs with DMSO (negative control), MCA/TPA (positive control) and MCA/TPA + 1 µM OSI-906 were performed until day 22. Afterwards 1.5 Mio cells/ml were applied into a clark-type electrode chamber and oxygen consumption was measured. Results indicated are mean + SD of 4 independent experiments with 4 technical replicates each. Statistical differences were calculated with a one-way ANOVA (post-hoc Scheffé) and appeared to be not significant.

**Supplement Figure 2**

**
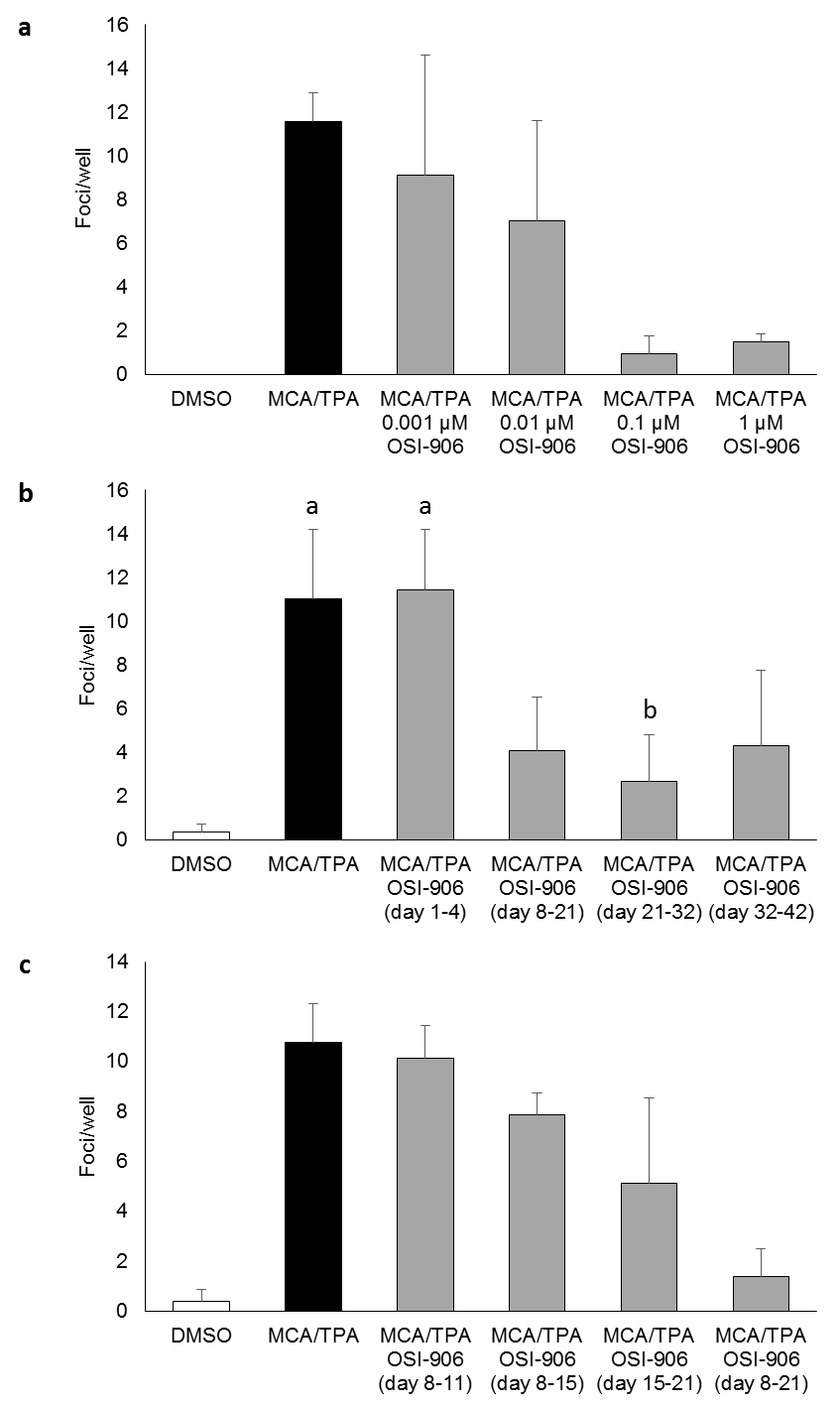

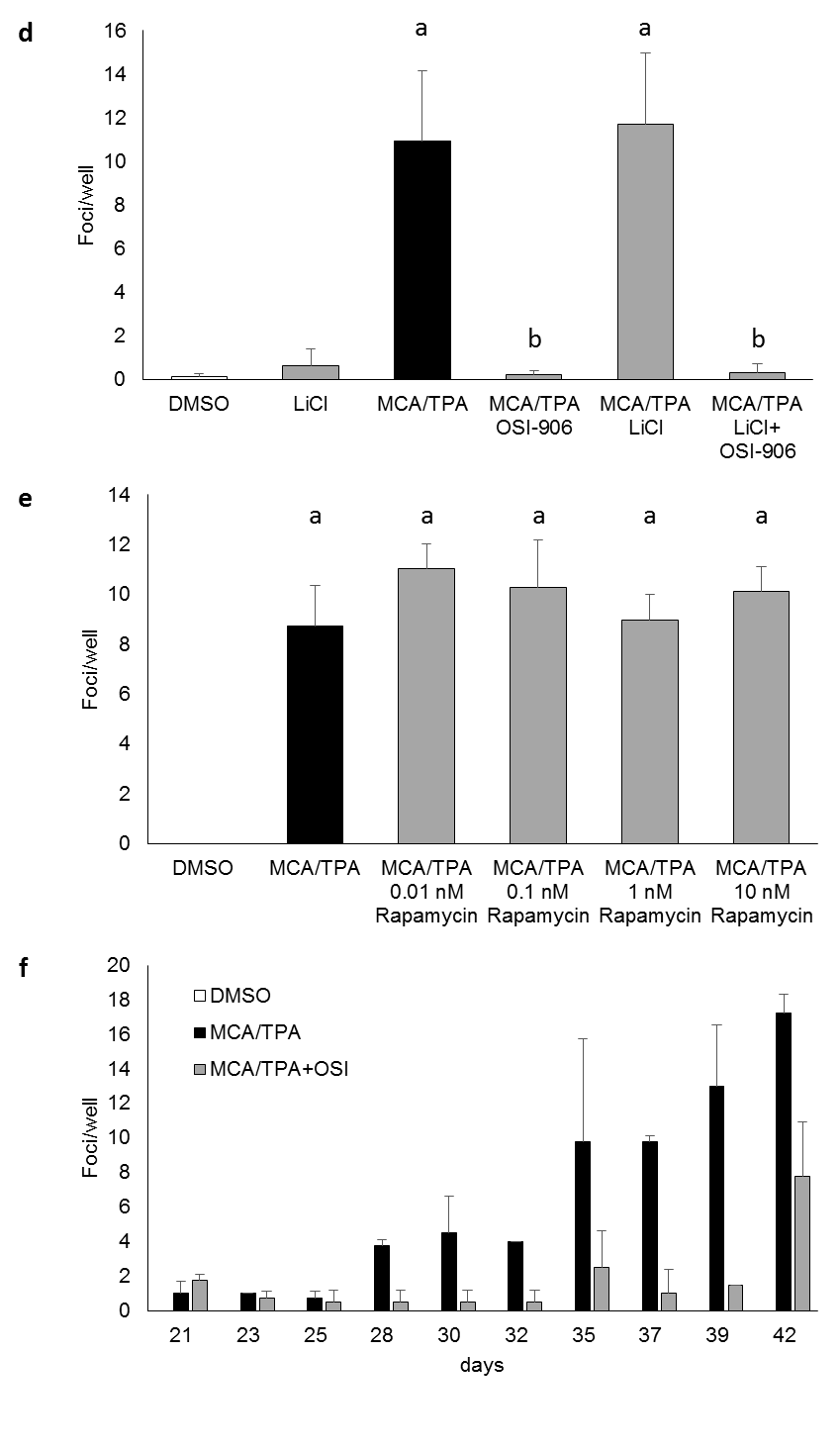
**

**Fig. S2: Analysis of transformed foci in the BALB-CTAs**

**a-f:** BALB-CTAs were performed with 0.5 µg/ml 3-Methylcholanthrene (MCA), 0.3 µg/ml 12‑O-Tetradecanoyl-phorbol-13-acetate (TPA), 3 mM LiCl and if not stated otherwise with 1 µM OSI-906. Two-stage transformation protocol was carried out until day 42 and afterwards cells were fixed and stained with Giemsa. Transformed type 3 foci were manually scored on the basis of the following criteria: (i) deep basophilic staining, (ii) spindle-shaped cells, (iii) multilayer growth, (iv) random cell orientation and (v) invasive growth of cells at the edge of foci. Foci with a diameter less than 2 mm were not included into the scoring. Results indicated are mean + SD of a single (c), two (a, f) or three (b, d, e) independent experiments with 4 replicates each. If possible, data were analyzed with a one-way ANOVA (post-hoc Bonferroni) and p‑values <0.05 were considered to be statistical significant (b, d, e).

^a^ Significantly different to the solvent control (DMSO)

^b^ Significantly different to the positive control (MCA/TPA)

**Supplement Figure 3**

**
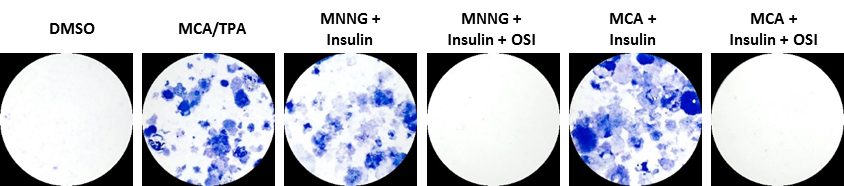
**

**Fig. S3: OSI-906 is also effective with chemicals other than MCA/TPA**

BALB-CTAs with DMSO (negative control), MCA/TPA (positive control) and the alternative initiator MNNG and promoter insulin were performed. Presented are selected pictures of 2 independent experiments. Additional treatment with 1 µM OSI-906 from day 1 to 42 in combination with these other initiators and promoters led also to no cell colony formation (blue colored).

**Supplement Figure 4**

**
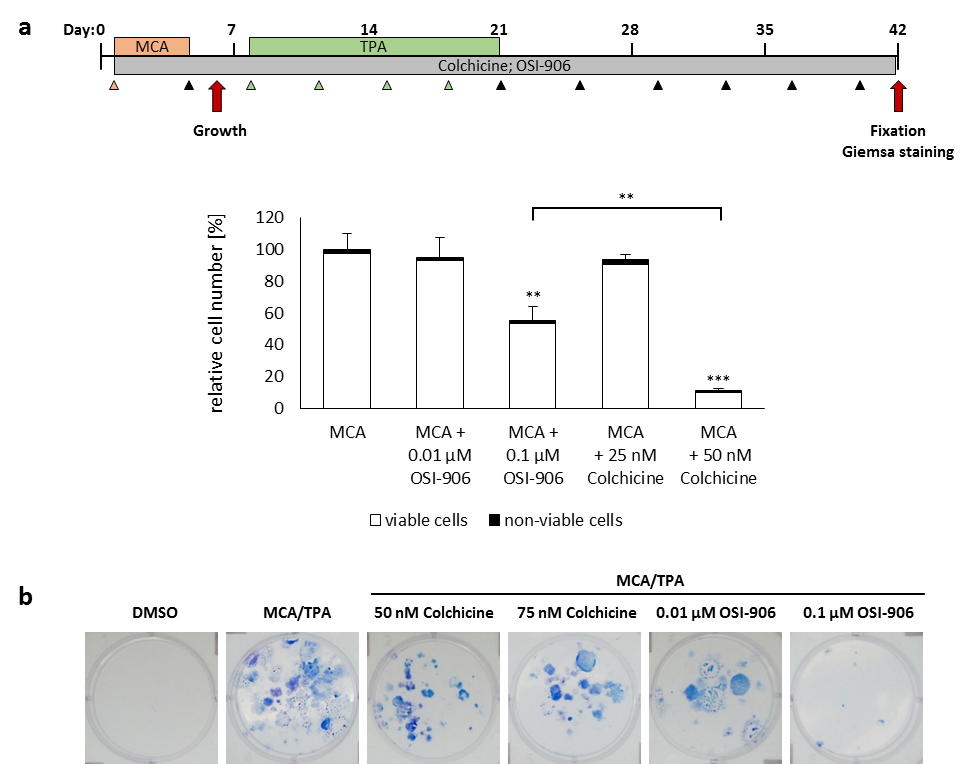
**

**Fig. S4: The growth inhibitory effect of OSI-906 is not causative for the inhibition of cellular transformation**

**a:** BALB/c cells were treated with MCA and OSI-906 or colchicine and counted on day 6 of the transformation assay. Results indicated are mean + SD of 4 replicates. Statistical differences are displayed as ** (p<0.01) and *** (p<0.001) according to a one-way ANOVA (post-hoc Dunnett T3). The lowest concentrations of OSI-906 and colchicine showed no growth inhibition in BALB/c cells. In contrast, 100 nM OSI-906 and 50 nM colchicine decreased cellular growth significantly, whereas 50 nM colchicine was more effective.

**b:** A BALB-CTA with different concentrations of OSI-906 or colchicine was performed until day 42. Presented are selected pictures of 4 replicates. Only 100 nM OSI-906 led to almost no cell colony formation, although 50 nM colchicine showed an even higher growth inhibition (as indicated in Fig. S4a).
